# The Effect of Emotional Closeness and Exchanges of Support Among Family Members on Residents’ Positive and Negative Psychological Responses After Hurricane Sandy

**DOI:** 10.1371/currents.dis.5eebc1ace65be41d0c9816c93d16383b

**Published:** 2016-08-24

**Authors:** Zhen Cong, Ali Nejat, Daan Liang

**Affiliations:** Civil, Environmental, and Construction Engineering, Texas Tech University, Lubbock, Texas

## Abstract

Introduction: This study examines how changes in emotional closeness and exchanges of support among family members after Hurricane Sandy affected residents' psychological outcomes both positively and negatively.

Methods: The working sample included 130 family ties reported by 85 respondents recruited from community and shelter residents on Staten Island after it was seriously damaged by the 2012 Hurricane Sandy. Regression with robust standard errors was used to examine how changes in emotional closeness and exchanges of support with adult family members affected respondents' posttraumatic psychological distress and posttraumatic growth.

Results: Results showed psychological distress was significantly increased with higher levels of instrumental support received from family members; whereas posttraumatic growth was significantly increased with greater improved emotional closeness with family members. In addition, having higher levels of education was associated with lower levels of psychological distress and respondents from shelters showed higher levels of posttraumatic growth than those who were from the community.

Discussion: It is suggested that after a significant disaster, although a family may be the best to take care of its members' emotional needs, it should not be expected to satisfy the instrumental needs of its members. In addition, posttraumatic psychological distress and growth are not necessarily opposite to each other; the psychological well- being of residents after a disaster needs to be carefully examined from both perspectives.

## Introduction

Natural disasters could result in various kinds of mental health problems 8. The adverse effects of disasters on mental health may include psychological distress and anxiety, a strong sense of loss, out of control, and feeling overwhelmed; some situations including physical injuries, witnessing death, missing family members, loss of property, loss of values and tradition, relocation, and lack of resources may add extra grief to the experience of disasters 2. These emotional reactions may lead to severe Post Traumatic Stress Disorder if not appropriately addressed.

Nonetheless, some survivors of disasters also demonstrated positive psychological gains. It is possible for survivors to experience positive change of perception of the self with a sense of increased personal strength, a changed sense of relationships with others with improved closeness of intimate relationships, and a changed philosophy of life with increased appreciation of life and setting new life priorities, which is conceptualized as post traumatic growth 6.

The stress process model indicates that social support will help to reduce people’s distress in times of high stresses 10. The disaster presents a highly stressful situation, where social support and individuals’ own psychological resources are tapped into to cope with the stresses. People respond to disasters as families, e.g., they usually evacuate together and most of the time continue their after-disaster life together and recover together. Support from families, especially the emotional support, tends to help people coping with stresses after disaster 4. However, few studies have differentiated types of support exchanged between family members. In addition, it is usually challenging to get an overall picture of support exchanged among a family with multiple ties included.

In this investigation, we examined both the positive and negative psychological outcomes after the 2012 Hurricane Sandy, which damaged more than a quarter-million residences and was responsible for around 150 direct deaths and 50 billion dollars losses 7. By considering both aspects of the psychological outcomes, it is possible to compare predictors for them side by side. Additionally, we examined how changes in emotional closeness and exchanges of emotional and instrumental support with all adult family members may affect respondents’ positive and negative psychological responses

## Methods

Sample

After the approval of Texas Tech University Human Research Protection Program, a survey was administered with residents of Staten Island in December 2012, approximately one month after the Hurricane Sandy hit the area. More than 50% of all deaths associated with Hurricane Sandy and over 30% of destroyed houses happened in Staten Island 12. In the year 2010, Staten Island had an population of about 468,000 with the median household income of $74,043 in 2014 dollar; the average household had 2.81 family members and 73% were White 13.The whole research process and used materials were approved by the Institutional Review Board of Texas Tech University. Participants were mainly recruited by door-to-door visits in heavily damaged areas, supplemented by residents of shelters hosting those who lost their homes and individuals who applied for assistance from FEMA disaster centers. Only respondents 18 years or older were interviewed. Altogether, 122 respondents were interviewed face-to-face. They also reported the household members that lived with them within one week before Sandy. Altogether, 198 household members were reported and among them 131 were aged 18 or above. We probed into respondents’ interactions with each adult member aged 18 and above after Sandy. Therefore, respondents who reported no adult family members were excluded from the study. After deleting missing values, the working sample includes 130 household members reported by 85 respondents.

Dependent Variables

K-6 measure was used to measure nonspecific psychological distress after Hurricane Sandy 3. Post Traumatic Growth was measured with the 10 item short form Post-Traumatic Growth Inventory scale 1.

Independent variables

To measure changes in emotional closeness after the hurricane, we asked respondents concerning each reported household member: How much did the hurricane bring you closer or less closer to this person? Answers were coded as 0 (a lot less closer) - 6 (a lot closer).

Emotional support received from household members was measured with a question “How much emotional support have you received from this person related to Sandy and its aftermath?”, and the answers were coded as 0 (none) - 4 (all you need). Emotional support provided as well as instrumental support received and provided were coded similarly. We gave examples of instrumental support as help with evacuation, transportation, looking for shelters, filling forms etc.

Controls

Variables controlled included respondents’ age, gender (0=male, 1=female), education (0=high school or less, 1= college or more), race (1=White, 0=others), and recruiting place (0=community, 1=shelters).

Analysis

Regression with robust standard errors was used to account for the non-independence of analytic units resulting from the clustering of reported ties within each respondent. We used STATA 12, “regress” command, and “robust” option for the analysis. With the “robust” option, the standard errors were estimated using the Huber-White sandwich estimators 14 .

## Results

Table 1 shows the coding and descriptives for analytic variables. Among the 85 respondents, 39% were female, 82% were white, 47% had college level or more education, and 39% were recruited from residents from shelters. The average age was 45.14 years (Standard Deviation (SD) = 10.66). On average, respondents showed some levels of psychological distress measured with K6 scale (M = 10.88, SD = 6.55) and posttraumatic growth (Mean (M) = 28.37, SD = 11.30). The mean K6 score was 10.88, lower than the severe mental distress cutoff point 13 but higher than the moderate distress cutoff point 5, representing moderate levels of distress after the hurricane 15 ^,^ 16. Concerning respondents’ changes in emotional closeness and support exchanges with the 130 adult family members, on average respondents reported slightly improved relationships with family members (M = 3.82, SD = 1.93). The average emotional support received from family members was 2.42 out of 4 (SD = 1.40), provided to family members was 2.80 out of 4 (SD = 1.35). Instrumental support received was 2.44 (SD = 1.51) and provided was 2.65 (SD = 1.47).

Table 2 shows that exchanges of support did not affect posttraumatic growth, but instrumental support received from family members increased psychological distress and emotional support received from family members reduced psychological distress. Having improved emotional closeness with family members did not affect psychological distress but significantly and considerably increased posttraumatic growth. Among controlling variables, higher education was associated with lower level of psychological distress, and respondents from shelters reported higher levels of posttraumatic growth.

**Table 1 Descriptives:** 

	Mean	Standard Deviation	Coding and Range
Respondents' characteristics (N=85)			
Psychological distress	10.88	6.55	0(no distress) - 24(most distress)
Post Traumatic Growth	28.37	11.30	0(no growth) - 50(most growth)
Age	45.14	10.66	23.5 years old - 71 years old
Gender			
Male	61.18%		
Female	38.82%		
Race			
White	82.35%		
Others	17.65%		
Education			
High school or less	52.94%		
College or more	47.06%		
Recruiting Places			
Community	61.18%		
Shelters (vs. community)	38.82%		
Damage levels			
Moderate or less damage	33.85%		
Severe	40.00%		
Destruction	26.15%		
Number of family ties reported			
1	56.47%		
2	30.59%		
3	7.06%		
4	3.53%		
Characteristics of family ties (N=130)			
Relationships with the respondent			
Spouse or partner	45.38%		
Child	22.31%		
Others	32.31%		
Age	40.19	15.26	18-83
Gender			
Male	47.69%		
Female	52.31%		
Changes in emotional closeness with family members	3.82	1.93^a^	0(a lot less closer) - 6(a lot closer)^d^
		1.83^b^	
		0.76^c^	
Emotional support received	2.42	1.40^a^	0(none) - 4(all the receiver needed)^e^
		1.24^b^	
		0.67^c^	
Emotional support provided	2.80	1.35^a^	0(none) - 4(all the receiver needed)^e^
		1.16^b^	
		0.67^c^	
Instrumental support received	2.44	1.51^a^	0(none) - 4(all the receiver needed)^e^
		1.25^b^	
		0.79^c^	
Instrumental support provided	2.65	1.47^a^	0(none) - 4(all the receiver needed)^e^
		1.28^b^	
		0.66^c^	
^**a**^ overall SD			
^**b**^ between respondents SD			
^**c**^ within respondents SD			
^**d**^ 0 (a lot less closer), 1 (somewhat less closer), 2 (only a little less closer), 3 (about the same), 4 (only a little closer), 5 (somewhat closer ), and 6 (a lot closer).			
^**e**^ 0 (none), 1 (a little bit), 2 (some), 3 (substantial), 4 (all s/he needs)			

Table 2 shows that exchanges of support did not affect post traumatic growth, but instrumental support received from family members actually increased psychological distress. Having improved emotional closeness with family members did not affect psychological distress but significantly and considerably increased post traumatic growth. Among controlling variables, higher education was associated with lower level of psychological distress, and respondents from shelters reported higher levels of post traumatic growth.

**Table 2 Regression predicting psychological distress and post traumatic growth with robust standard errors (N=130 ties from 85 respondents):** 

	Psychological Distress	Post-Traumatic Growth
Age	-0.01	-0.04
Female (vs. male)	1.68	1.10
White (vs. others)	0.55	-3.74+
College or more education (vs. high school or less)	-2.40*	-1.11
Shelter (vs. community)	-1.62	6.53**
Damage (reference: moderate or less damage)		
Severe	5.85**	-2.30
Destruction	3.62*	-3.70
Changes in emotional closeness with family members	0.23	2.36***
Emotional support received	-2.03**	-0.02
Emotional support provided	0.83	-1.05
Instrumental support received	1.88**	1.01
Instrumental support provided	-0.74	-0.92
Constant	7.27	25.44
R^2^	0.29	0.23
+p<0.1, *p<0.05, **p<0.01, ***p<0.001		

## Discussion

This study examines how changes in emotional closeness and exchanges of support with all adult family members affected both the positive and negative dimensions of psychological responses after a significant disaster. It is interesting that instrumental support received from family members increased psychological distress and improved emotional closeness with family members increased post traumatic growth. Although it is generally expected that social support will be beneficial for individuals’ psychological well-being, previous literature has shown possible negative effects of social support. For example, the task specific theory proposes that the best psychological outcomes will occur when there is a good match of providers of support and the tasks of support 5. Based on the findings, we suspect that family members are not good providers of instrumental support such as transportation and evacuation during the highly stressful period after a disaster and when family members are sharing similar needs. Under such circumstances, receiving instrumental help may bring a sense of guilty as it may put an obvious burden on the ones who provided support, which will eventually leads to elevated psychological distress. Consequently, help with transportation and evacuation etc. should be more effective when it comes from formal social services. But our findings did shown the beneficial effects of emotional support from family members.

At the same time, we find that post traumatic growth and post traumatic distress were predicted by different factors. The findings show that higher levels of education were associated with lower levels of psychological distress, but not necessarily post traumatic growth. Similarly, dwelling in shelters enticed a stronger sense of post traumatic growth, but did not reduce psychological distress. Similarly, severe damage to houses increased distress but did not affect posttraumatic growth. Previous literature has shown that post traumatic growth does not mean that individuals could potentially recover to the pre-disaster status, but suggests that this traumatic experience could serve as an opportunity for development; it is different from reduced stress or increased well-being, and it is not the opposite of the psychological distress 11. Individuals could learn from the disaster when they were trying to cope with the events; they will possibly find their own strength that they did not realize in the past and are more prepared and determined to deal with future hardships and difficulties 9. This may explain why residents who stayed in shelters who typically experienced more losses and stresses showed greater post traumatic growth than community dwelling residents did.

This study has a relatively small sample size, which limits the statistical power of the analysis and the potential to include more controlling variables. Furthermore, because this study is cross-sectional, the findings of this study speak more about the association between independent and dependent variables rather than argue for any causal relationship. Additionally, we relied on respondents’ own report of their interactions with family members. The self-reported measures might be biased, as that respondents tended to report higher levels of support provided than received. But the gap was not huge as shown in this study, which suggested limited biases in the self-reported exchanges of support. Finally, we were not able to include interactions among all family members, which would contribute to a more comprehensive understanding of family interactions after a disaster.

Despite all those limitations, this study includes both the positive and negative dimensions of psychological responses and tries to include a comprehensive picture of exchanges of support within a family. The implications for policy makers include carefully weighing the benefits of family support and thus make decisions on providing formal support to maximize the psychological well-being of residents who experienced significant stresses in a disaster.

## Data Availability

Data is available from Figshare at https://dx.doi.org/10.6084/m9.figshare.3600321.v1. The DOI is “10.6084/m9.figshare.3600321”.
